# In Older Adults, Perceived Stress and Self-Efficacy Are Associated with Verbal Fluency, Reasoning, and Prospective Memory (Moderated by Socioeconomic Position)

**DOI:** 10.3390/brainsci12020244

**Published:** 2022-02-10

**Authors:** Ulrike Rimmele, Nicola Ballhausen, Andreas Ihle, Matthias Kliegel

**Affiliations:** 1 Swiss National Centre of Competence in Research LIVES–Overcoming Vulnerability: Life Course Perspectives, University of Geneva, 1205 Geneva, Switzerland; N.M.Ballhausen@tilburguniversity.edu (N.B.); andreas.ihle@unige.ch (A.I.); matthias.kliegel@unige.ch (M.K.); 2 Center for the Interdisciplinary Study of Gerontology and Vulnerabilities, University of Geneva, 1205 Geneva, Switzerland; 3 Emotion and Memory Laboratory, Department of Psychology, FPSE, University of Geneva, 1205 Geneva, Switzerland; 4 Swiss Center for Affective Sciences, University of Geneva, 1205 Geneva, Switzerland; 5 Geneva Neuroscience Center, University of Geneva, 1211 Geneva, Switzerland; 6 Department of Developmental Psychology, Tilburg University, 5037 AB Tilburg, The Netherlands; 7 Cognitive Aging Lab, Department of Psychology, FPSE, University of Geneva, 1205 Geneva, Switzerland

**Keywords:** older adults, perceived stress, self-efficacy, socioeconomic position, cognitive performance, prospective memory

## Abstract

Despite evidence that stress relates negatively to cognitive functioning in older adults, little is known how appraisal of stress and socioeconomic meso-level factors influence different types of cognitive functions in older adults. Here, we assess the relationship between perceived stress (PSS scale) and a battery of cognitive functions, including prospective memory in 1054 older adults (65+). A moderator analysis assessed whether this relationship varies with neighborhood socioeconomic status using an area-based measure of Socioeconomic Position (SEP). Perceived stress was associated with worse processing speed, verbal fluency, and inductive reasoning. The perceived self-efficacy subscale of the PSS is related to better performance in these measures. Higher self-efficacy was also associated with better prospective memory; this relationship was more pronounced for people with high neighborhood SEP. These findings indicate that not only do perceived stress and perceived self-efficacy relate to cognitive functioning in older age but also that neighborhood SEP is a moderator of this relationship.

## 1. Introduction

Evidence from experimental studies indicates that stress modulates cognition, with the brain being most sensitive to stress during childhood and older age [1,2]. As such, stress may represent a risk factor contributing to age-related cognitive decline [2]. Indeed, population-based studies showed that increased levels of perceived stress relate to lower cognitive functioning and a faster rate of cognitive decline [3,4,5,6,7]. Furthermore, enhanced perceived stress levels predict the transition from normal cognition to amnestic mild cognitive impairment and from amnestic mild cognitive impairment dementia [8,9].

Thereby, the effects of stress on cognition vary depending on the appraisal of stressful events and the type of cognitive function under study [10,11]. Appraisal of stressful events is assessed with the perceived stress scale (PSS) [12]. This questionnaire contains two subscales assessing different properties of stress [13,14,15]. The positively worded subscale (PSS-PW) is thought to measure perceived coping abilities such as self-efficacy, while the negatively worded subscale (PSS-NW) measures perceived helplessness. These two subscales are poorly correlated in older adults [16] and relate differently to cognitive functions [17]. Crucially, the self-efficacy subscale (PSS-PW scale), but less so the helplessness subscale (PSS-NW), is associated with cognitive functioning in older adults [18,19,20]. Moreover, people with high self-efficacy show lower stress reactivity [21].

Concerning the type of cognitive function, memory performance in episodic or semantic memory tasks relates negatively to the PSS sum score [4,22]. In contrast, the PSS-PW subscale relates positively to performance in various cognitive functions in older adults, such as processing speed, memory, and executive functions [19,20].

Stress-related effects on an additional cognitive domain, prospective memory, have only recently received more attention. Prospective memory refers to the capacity to remember to carry out an intended action at an appropriate point in the future while being engaged in an ongoing activity [23,24]. As such, prospective memory is highly relevant for the maintenance of one’s functional independence and autonomy across the life span [25]. The first experimental studies indicate that acute stress affects prospective memory in young adults [26]. On the contrary, in older adults, studies have not shown a clear link between stress and PM performance [27,28]. As sample sizes were small in these studies, it remains unclear how stress might influence prospective memory in older age.

Hence, the first aim of our study was to examine the relationship between perceived stress and cognitive performance in a comprehensive battery of cognitive tests, including prospective memory, testing a large sample of older adults. Hereby, we aimed to replicate previous findings showing that perceived stress, particularly the PSS-PW self-efficacy subscale, relates to multiple domains of cognitive functioning. Furthermore, we examined how perceived stress relates to prospective memory.

In a recent conceptual framework on life course stress effects and vulnerability in old age, we suggested that (cognitive) vulnerability in older age may stem from a lack of sufficient reserves across multiple domains, notably including social and economic resources [29]. In that context, we argued that, besides cognitive mechanisms, it is important to understand how socioeconomic reserves, which are built up over the life course, may affect cognition in older age (see [30,31]) for testing how social reserves affect cognitive aging in general). One important proxy of socioeconomic reserves is socioeconomic status, which includes indicators such as education, occupation, and income [32]. Interestingly, socioeconomic status relates to stress reactivity and cognitive function [33,34] and, therefore, might be a potential moderator for the relationship between perceived stress and cognitive functioning. To examine the relationship between socioeconomic status and stress reactivity, previous studies mostly used indices that focused on the individual level, such as income or the educational attainment of an individual [35,36,37].

More recently, it was argued that important additional information is captured when meso-level context factors are taken into account, and socioeconomic position (SEP) is assessed at a community or area level [38]. Indeed, neighborhood characteristics are shown to contribute to mental health and cognitive functioning in older adults [39,40]. This relationship is of particular importance for older adults given that the activity spaces of older adults become increasingly restricted to their immediate surroundings due to limited mobility or functional decline, therefore making them particularly vulnerable to neighborhood stressors [41,42]. Yet the moderating role of neighborhood socioeconomic position between stress and cognitive functioning was not previously examined. Hence, the second aim of our study was to examine whether neighborhood SEP, assessed as an area-based measure, moderates the relationship between perceived stress and cognitive performance in older adults.

## 2. Materials and Methods

### 2.1. Study Participants

The Vivre–Leben–Vivere study is a large interdisciplinary survey of the life and health conditions of older adults living in Switzerland [43,44,45]. The main sample of the Vivre–Leben–Vivere study was randomly selected from the cantonal and national population records and stratified by age (65–69, 70–74, 75–79, 80–84, 85–89, 90, and above) and sex [44]. The second wave of the Vivre–Leben–Vivere study study conducted in 2017 included measures of cognitive functions and perceived stress assessed via self-administered questionnaires and face-to-face interviews in 1059 participants. From this sample, we excluded 16 participants because an interviewer did not follow the study protocol and another 15 participants had a score below 21 in the Mini-Mental State Examination [46]. Given that we assessed cognitive variables in the present paper, we excluded 15 participants with a score below 21 in the Mini-Mental State Exam, which corresponds to 1.42% of the full sample (N = 1059). We chose a cut-off for a score of 21 points, as participants with dementia have scores below 21 in the Mini-Mental State Exam [46]. All participants of the final sample (N = 1028) completed the prospective memory task. Due to a random rotation procedure, two-thirds of the 1028 participants, i.e., 683 participants, completed the cognitive tests of the Cognitive Telephone Screening Instrument (COGTEL; Table 1). Power analysis with Gpower [47] showed that in order to obtain an effect size of f^2^ = 0.032, similar to previous studies examining the relationship between perceived stress and cognitive functions [22], with 80% power using linear regression (alpha = 0.05), a sample size of 245 participants would be needed.

All participants provided written informed consent before participation. The study protocol was in accordance with the Declaration of Helsinki and was approved by the local ethics committee.

### 2.2. Measures

Perceived Stress was assessed with the 4-item version of the Perceived Stress Scale (PSS). The PSS measures how stressful individuals appraise situations in their life that occurred during the previous month [12]. The 4-item version contains two positively worded items (e.g., “In the last month, how often have you felt confident about your ability to handle your personal problems?”) and two negatively worded items (e.g., “In the last month, how often have you felt that you were unable to control the important things in your life?”). Responses were provided to every item using a 5-point Likert scale ranging from never (0) to very often (4). Summing the negatively and the positively worded items separately resulted in two subscales that were suggested to measure different properties of stress, i.e., perceived helplessness/distress subscale (PSS-NW) and the perceived self-efficacy/coping subscale (PSS-PW). Both subscales ranged from 0–8; higher scores indicate greater perceived helpless-ness/distress and self-efficacy/coping, respectively [16]. After reversing the positively worded items’ scores, responses to all items were summed, resulting in an overall perceived stress score (range 0–16; higher total scores indicating greater perceived stress).

### 2.3. Cognitive Functioning

Face-to-face interviews assessed cognitive functioning with the Mini Mental State Exam, the Trail Making Test Part A and B (TMT A and TMT B), prospective memory, and the six subtests of the Cognitive Telephone Screening Instrument (COGTEL) [48,49].

The Mini Mental State Exam assesses global cognitive functioning and is commonly used as a screening tool for cognitive impairment [50].

The Trail Making Test (TMT) consists of two parts, which measure processing speed (TMT A) and cognitive flexibility (TMT B). For TMT A, participants were instructed to connect a sequence of numbers from 1 to 25 as fast as possible without errors, after practicing the task once with a sequence from 1 to 8. To measure cognitive flexibility with TMT B, participants were given one practice trial alternating connecting numbers and letters, i.e., connecting 1-A-2-B-3-C-4-D. They were then instructed to alternate in connecting the numbers 1 to 13 in ascending and the letters A to L in alphabetic order, i.e., 1-A-2-B-3-C until 12-L-13, without making any error [51].

Prospective memory was assessed with four event-based tasks, i.e., a task in which the execution of the intended action is triggered by the presentation of a specific external target event. At the beginning of the interview, the experimenter instructed the participants to be unsolicited (1) say red pen at the time of the interview that the interviewer talked about a red pen, (2) knock twice on the table when the interviewer talked about physical activities, (3) tell the interviewer the year of birth when talking about activities in the course of the participant’s life, and (4) remind the interviewer to switch on their mobile phone at the end of the interview.

The COGTEL assesses verbal short-term and long-term memory, working memory, verbal fluency, inductive reasoning, and prospective memory.

For verbal short-term and long-term memory, participants learned four pairs of un-related words read aloud for 3 s each. After a short break, the experimenter cued the participants with the first words, in a different order than the word pairs had been learned. Participants recalled the second pair of words in the following 5 s (short-term memory scores ranging from 0 to 4) [46]. For correct responses, the experimenter gave positive feedback; for incorrect responses, the experimenter repeated the correct word for later assessment of long term memory. Three COGTEL subtests (i.e., working memory, verbal fluency and inductive reasoning) were then interspersed between the immediate and the delayed recall of the word pairs. For the long-term memory test, the experimenter provided the first word again and asked participants to recall the second word of each pair in the 10 s after the cue word (verbal long-term memory score ranging from 0 to 4).

Working memory was assessed with the backward digit span test of the WAIS-R [52]. Participants had to recall a list of digits in reverse order of progressively longer sequences of single-digit numbers presented at a 1-s rate. The test was stopped if participants failed two trials at a given sequence length [46,49]. Scores ranged from 0 to 12, indicating the number of correctly recalled sequences.

Verbal fluency was assessed with a letter fluency and a category fluency task [53]. Participants were given 60 s to produce as many words beginning with the letter A and 60 s to produce as many professions as possible. The letter and category fluency scores were summed to a total verbal-fluency score.

Inductive reasoning was assessed with a subtest of the WAIS-R [52]: Participants had to add a sixth number to a sequence of five numbers following specific rules that they had to detect themselves. The test was stopped if participants failed two trials at a given sequence. Scores range from 0 to 8, representing the number of correctly completed sequences out of the eight given sequences.

### 2.4. Neighborhood Socioeconomic Position

As an indication of socioeconomic position, we used the Swiss neighborhood index of socioeconomic position (Swiss-SEP). The Swiss neighborhood SEP is an area-based measure of socioeconomic position based on data on income, education, occupation, and housing conditions obtained from the 2000 census and provided by the Swiss National Cohort (SNC) [54]. The Swiss neighborhood SEP ranges from 0 (lowest neighborhood SEP) to 100 (highest neighborhood SEP), with a median of 63.32.

### 2.5. Statistical Analyses

**Figure 1:**
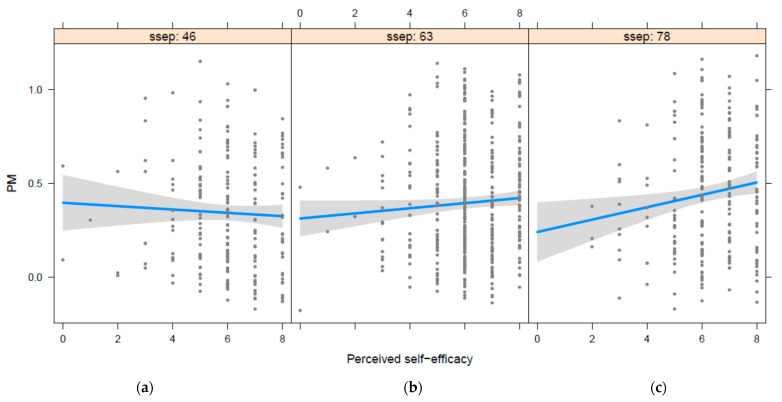
The relation between perceived self-efficacy (scale 0–8) and prospective memory is moderated by socioeconomic position (SSEP). On the left panel (**a**) is low SSEP (46), in the middle panel (**b**) medium SSEP (63), and on the right panel (**c**) high SSEP (78).

Mean scores and standard deviations were computed for age, years of education, perceived stress (PSS and its subscales), and all cognitive tests. Two-tailed t-tests specified whether the subgroup of participants that underwent the COGTEL differed from the one-third of participants that did not undergo the COGTEL. Bivariate correlations between age, education, perceived stress, and all domains of cognitive functions and neighborhood SEP were computed. Linear regression analyses were performed separately for the PSS-PW subscale, the PSS-NW subscale, and the total PSS scales as independent variables, and the different cognitive measures as the dependent variable. All regression analyses controlled for age, sex, and years of education. Last, we examined whether the relationship of perceived stress (and PSS subscales) on cognition varied as a function of socioeconomic position. To do so, we followed the guidelines by Hayes and used the PROCESS macro in SPSS to test for moderation effects [55]. Specifically, using SPSS PROCESS, we regressed perceived stress (and PSS subscales) on cognitive performance (controlling for age, sex, and education), including neighborhood socioeconomic position as moderator plus an interaction term. We then used the statistical package R for visualization of the moderator results in Figure 1.

## 3. Results

### 3.1. Sample Characteristics

The sample is described in Table 1. On average, the 1028 participants (508 female) were 80.68 years (SD = 6.65) old and had followed 13.32 years of education (SD = 4.08). The subsample that did the COGTEL consisted of 683 participants (342 female, i.e., 49.93% female) with a mean age of 80.46 years and 13.39 years of education. The COGTEL subsample did not differ from the participants without COGTEL in age, years of education, perceived stress, TMT A and TMT B, prospective memory, and neighborhood SEP (all ps > 0.06). Of note, the measurements of some participants were missing. In addition, the measurements of some participants were missing due to refusals or technical problems, i.e., 6.00% of the 683 participants did not have a valid short-term memory score (N = 41), 7.32% had no scores for long-term memory (N = 50), 1.17% participants were missing score a for working memory (N = 8), and 1.17% were missing a score for inductive reasoning (N = 8). Table 1 indicate the number of participants included for each measure.

Authors should discuss the results and how they can be interpreted from the perspective of previous studies and of the working hypotheses. The findings and their implications should be discussed in the broadest context possible. Future research directions may also be highlighted.

### 3.2. Correlations between Age, Education, PSS, Neighborhood SEP, and Cognitive Functions

Age and education correlated significantly with perceived stress and cognitive functions. In particular, higher age was correlated with lower cognitive functioning (all *p* < 0.001), higher values of PSS sum score (*p* < 0.001), PSS-NW subscale (*p* < 0.01), and lower values of the PSS-PW scale (*p* < 0.001). Years of education correlated positively with all measures of cognitive functions (*p* < 0.001) other than TMT A short-term and long-term memory (all *p* > 0.12). Higher education was related to a lower PSS sum score (*p* < 0.05) and a higher score on the self-efficacy scale (*p* < 0.05).

The subscales PSS-PW and PSS-NW were negatively correlated (r = −0.45, *p* < 0.001). The total score of the PSS correlated negatively with performance in the COGTEL tests for working memory, inductive reasoning, verbal fluency, and prospective memory, but negatively with performance in TMT A and TMT B. Similarly, the PSS-NW scale was correlated negatively with COGTEL tests for inductive reasoning and verbal fluency, but positively with TMT A and B. In contrast, the PSS-PW scale was correlated positively with Mini-Mental State Exam scores and all cognitive measures of the COGTEL (all *p* < 0.01), except for short-term memory (*p* = 0.065), but negatively with TMT A and TMT B (both *p* < 0.01, see Table 2 for correlations of PSS/PSS subscales with cognitive functions).

In addition, PSS sum score (r = 0.09, *p* < 0.05) and PSS-PW were correlated with neighborhood SEP (r = 0.09, *p* < 0.05), but not PSS-NW scale (*p* > 0.05). Neighborhood SEP was correlated with the Mini-Mental State Exam (r = 0.12, *p* < 0.01), working memory (r = 0.10, *p* = 0.01), and prospective memory (r = 0.13, *p* < 0.01), but no other cognitive variables (all *p* > 0.11).

### 3.3. Regression Analyses

Table 3 and Table 4 show linear regression results between the total perceived stress (the PSS subscales) scale and cognitive measures. Perceived stress was related to worse global cognition assessed with the Mini-Mental State Exam, slower processing speed/flexibility (TMT A/ TMT B), lower verbal fluency, and inductive reasoning. In contrast, the subscale perceived self-efficacy related to better performance in these measures, except for TMT B. In addition, perceived self-efficacy was related to better prospective memory. The perceived helplessness subscale related to only one cognitive outcome measure, i.e., to reduced performance in the TMT A (processing speed) and TMT B (flexibility).

### 3.4. Moderation Analyses

Neighborhood SEP moderated the relationship between perceived self-efficacy and prospective memory (Table 5). For individuals with high neighborhood SEP there was a significant positive relationship between perceived self-efficacy and prospective memory, e.g., neighborhood SEP = 78, β = 0.033, *p* < 0.01. This relationship was not significant in individuals with neighborhood SEP in the low and middle range (for median neighborhood SEP = 63, β = 0.013, *p* = 0.07; low neighborhood SEP = 46, β = −0.0098, *p* = 0.426; Figure 1).

Neighborhood SEP did not moderate the relationship between the PSS sum score (the PSS-NW subscore) and the cognitive tests of COGTEL and TMT.

## 4. Discussion

This population-based cross-sectional study examined how perceived stress (PSS questionnaire) relates to cognitive functioning in older adults (>65 years). Perceived stress was related to decreased global cognition assessed with Mini-Mental State Exam, slower processing speed/flexibility assessed with TMT, and poorer verbal fluency and inductive reasoning assessed with COGTEL. These results were mainly driven by lower perceived self-efficacy (PSS-PW subscale of the PSS). In particular, the PSS-PW scale was positively related to global cognition, verbal fluency, inductive reasoning, and reduced processing speed. Furthermore, perceived self-efficacy was positively related to prospective memory. Importantly, neighborhood socioeconomic position (SEP) moderated the relationship between perceived self-efficacy and prospective memory. Perceived self-efficacy had a more pronounced positive impact on prospective memory in people with high neighborhood SEP than those with low and medium neighborhood SEP.

The finding of an association between perceived stress and global cognition, processing speed, and verbal fluency in old adults is consistent with previous findings [4,7,19,20,58]. However, in contrast to other studies, we found no association between perceived stress and short term and long term verbal memory. One potential reason for this discrepancy is that we did not obtain sufficient variability in our memory test as it consisted of only four word pairs in contrast to previous studies that used more difficult memory tests [4,22]. In particular, VonDras et al. [22] found perceived stress to be associated with memory for complex prose passages but not with memory for verbal paired associates. This result suggests that stress has a greater impact on memory tasks with strong demand on executive functioning. Along this line, our findings suggest that increased levels of perceived stress contribute to poorer cognitive performance in tasks requiring greater executive resources, such as inductive reasoning.

Considering the two subscales of the PSS, we found that the perceived self-efficacy subscale PSS-PW and the perceived helplessness subscale PSS-NW were moderately correlated (r = −0.45, *p* < 0.001). This finding is in accordance with previous findings [20] and suggests that although there are commonalities between these two factors, there are also differences. Crucially perceived self-efficacy was associated with better cognitive performance in multiple cognitive tests, while perceived helplessness was associated only with slower processing speed. Previous findings likewise showed that the subscale perceived self-efficacy is related to cognitive functions rather than the subscale perceived helplessness [19,20]. In particular, in our study, increased self-efficacy was related to better performance in the Mini-Mental State Exam, TMT, verbal fluency, and inductive reasoning, as well as prospective memory. To the best of our knowledge, this is the first population-based study discovering a positive relationship between perceived self-efficacy and prospective memory in older adults. This result links an additional cognitive function, i.e., prospective memory, to perceived self-efficacy extending previous findings that reported perceived self-efficacy to relate to multiple domains of cognitive functions in older age [19,20].

Interestingly the relationship between perceived self-efficacy and prospective memory was moderated by neighborhood SEP: The positive relationship between perceived self-efficacy and prospective memory was only reliable for older adults with high neighborhood SEP, while this relationship was eliminated in older adults with low and medium neighborhood SEP. A link between perceived control and socioeconomic status was well-established in previous studies, with people with higher socioeconomic status displaying higher perceived control [59,60,61]. Similarly, a median-split in our study showed that participants with high SEP showed marginally higher self-efficacy (M = 6.20, SE = 0.07) than participants with low SEP (M = 6.02, SE = 0.06, F(1,888)= 3.565, *p* = 0.059), although SEP and self-efficacy were not strongly correlated (r = 0.082, *p* = 0.015). A potential explanation for our novel finding that perceived self-efficacy and prospective memory was moderated by neighborhood SEP is that adverse socioeconomic neighborhood conditions are less likely to enable older adults to have sufficiently frequent experiences of successful coping with stressful events, especially in relation to cognitive challenges under stress, which would allow building up of higher levels of self-efficacy. This might be of particular significance for prospective memory tasks, in which the challenge is to self-initiate an intended action at the right target moment without being prompted by the experimenter. With a build-up of higher self-efficacy through high neighborhood SEP, these participants might have more successfully coped with the key challenge of a prospective memory task (e.g., fewer doubts whether they face the right target moment or not and thus more frequent self-initiation of the intended action).

Future studies should test whether improving perceived self-efficacy in older age could be a means of reducing perceived stress, especially in the lower SEP groups, and thus maintaining prospective memory function. Such an intervention may potentially be effective, given that previous studies have demonstrated that experimental interventions can result in changes in one’s perceived level of control, suggesting that perceived self-efficacy is modifiable [62]. Other studies have directly modified the level of self-efficacy [63,64]. Along this line, it was shown that stress mindsets are related to perceived stress [65].

A limitation of the present study is that we took cross-sectional data from the Vivre–Leben–Vivere 2 survey. As such, we are unable to determine the causality for the relationship between perceived stress and cognitive function. In addition, only one measure of perceived stress was acquired. The PSS questionnaire assesses stress perceived within the month prior to the survey but does not ask for intensity or duration of stressors. Consequently, we are not able to assess the relationship between chronic stress and cognitive function, nor cognitive decline. In addition, our effects are small to moderate, yet in a similar range as previous findings [4,5,20]. Despite these limitations, our study has several strengths. Firstly, we obtained data from a large sample of older adults. Secondly, we assessed multiple cognitive variables, one of them, prospective memory, for the first time. Last, we considered important confounding factors, such as age, gender, and education.

## 5. Conclusions

Our data from a large population-based sample provides important scientific evidence for an association between perceived stress/perceived self-efficacy and cognitive functioning in older adults. For our prospective memory, this relationship is moderated by neighborhood SEP. Understanding how perceived stress/perceived self-efficacy is related to cognition is crucial for future interventions studies that should test whether altering perceived stress/perceived self-efficacy levels could help to prevent cognitive decline, an issue of major individual and public health importance.

## Figures and Tables

**Table 1:** Descriptives of the sample.

	N	Mean (SD)
Age	1028	80.68 (6.65)
Female	508	
Education (years)	1028	13.32 (4.01)
Perceived Stress Scale (PSS) total	955	3.95 (2.51)
PSS positive	956	6.10 (1.43)
PSS negative	956	2.10 (1.55)
Mini Mental State Exam	1028	28.26 (1.93)
TMT A (in sec.)	863	56.03 (24.37)
TMT B (in sec.)	438	108.90 (45.40)
COGTEL		
Short-Term Memory	642	0.92 (1.00)
Long-Term Memory	633	1.63 (1.25)
Working Memory	675	5.27 (1.80)
Verbal Fluency	683	24.55 (8.42)
Inductive Reasoning	675	3.06 (1.82)
Prospective Memory	1028	0.35 (0.33)
Swiss Social Economic Position Index (SEP)	940	62.63 (12.11)

**Table 2:** Pearson’s correlations between perceived stress and cognitive functions.

	Perceived Self-Efficacy (PSS-PW)	Perceived Helplessness (PSS-NW)	Sum Score PSS (PSS-Total)
Mini Mental State Exam (MMSE)	0.196 *****	−0.028	−0.109 ****
TMT A (in s)	−0.178 *****	0.172 *****	0.195 *****
TMT B (in s)	−0.143 ***	0.110 *	0.156 ***
COGTEL			
Short-Term Memory	0.075	−0.069	−0.062
Long-Term Memory	0.120 ***	−0.040	−0.068
Working Memory	0.105 **	−0.054	−0.085 *
Verbal Fluency	0.192 *****	−0.081 *	−0.142 *****
Inductive Reasoning	0.219 *****	−0.104 **	−0.180 *****
Prospective Memory (PM)	0.128 *****	−0.050	−0.083 *

* *p* < 0.05, ** *p* < 0.01, *** *p* < 0.005, **** *p* < 0.001, ***** *p* < 0.0001. Pearson’s correlation coefficients of 0.10, 0.30 and 0.50 are thought to represent a weak, medium and strong effects, respectively according to Cohen’s recommendation [56]. According to a quantitive investigation, normative guidelines for small, medium, and large true score correlations are suggested to be 0.15, 0.25, and 0.35 [57].

**Table 3:** Linear regression results between total perceived stress, the perceived self-efficacy subscale (PSS-PW), the perceived helplessness scale (PSS-NW) and the Mini-Mental State Exam (MMSE), processing speed (TMT A), and flexibility (TMT B).

	MMSE	TMT A	TMT B
	β	β	β
Perceived Stress Sum Score	−0.090 **	0.151 *****	0.101 *
Age	−0.114 *****	0.320 *****	0.397 *****
Education	−0.106 *	−0.007	−0.042
Sex	−0.059	0.035	−0.021
R^2^	0.04	0.142	0.186
Perceived Self-Efficacy (PSS-PW)	0.176 ****	−0.120 *****	−0.043
Age	−0.096 ***	0.319 *****	0.400 *****
Education	0.101 ***	−0.009	−0.042
Sex	−0.062	0.031	−0.024
R^2^	0.061	0.133	0.178
Perceived Helplessness (PSS-NW)	−0.015	0.143 ***	0.101 ^*^
Age	−0.126 *****	0.322 *****	0.393 *****
Education	0.107 ***	−0.014	−0.064
Sex	−0.052	0.037	−0.014
R^2^	0.032	0.137	0.176

* *p* < 0.05, ** *p* < 0.01, *** *p* < 0.005, **** *p* < 0.001, ***** *p* < 0.0001.

**Table 4:** Linear regression results between total perceived stress, the perceived self-efficacy subscale (PSS-PW), the perceived helplessness scale (PSS-NW), and the different cognitive measures.

COGTEL	Short-Term Memory	Long-Term Memory	Working Memory	Verbal Fluency	Inductive Reasoning	Prospective Memory
	β	Β	Β	β	β	β
Perceived Stress Sum score	−0.032	−0.020	−0.055	−0.085 *	−0.141 ****	−0.034
Age	−0.204 *****	−0.290 *****	0.080 *	−0.225 *****	−0.118 ***	−0.267 *****
Education	0.026	0.015	0.147 *****	0.191 *****	0.149 ****	0.143 *****
Sex	−0.103 *	−0.057	0.014	−0.042	0.057	0.037
R^2^	0.058	0.092	0.038	0.115	0.078	0.108
Perceived Self-Efficacy (PSS-PW)	0.030	0.058	0.073	0.126 ***	0.180 *****	0.071 *
Age	−0.203 *****	−0.280 *****	−0.072	−0.210 *****	−0.102 **	−0.260 *****
Education	0.026	0.013	0.146 *****	0.189 *****	0.147 ****	0.141 *****
Sex	−0.100 *	−0.055	0.018	−0.037	0.061	0.036
R^2^	0.058	0.094	0.041	0.12	0.089	0.107
Perceived Helplessness (PSS-NW)	−0.058	−0.014	−0.032	−0.046	−0.074	−0.016
Age	−0.204 *****	−0.292 *****	−0.086 *	−0.235 *****	−0.136 ****	−0.272 *****
Education	0.026	0.016	0.149 *****	0.194 *****	0.154 ****	0.141 *****
Sex	−0.107 *	−0.057	0.015	−0.041	0.059	0.040
R^2^	0.061	0.092	0.036	0.11	0.064	0.107

* *p* < 0.05, ** *p* < 0.01, *** *p* < 0.005, **** *p* < 0.001, ***** *p* < 0.0001.

**Table 5:** Interaction of perceived stress and SEP on prospective memory.

	Prospective Memory B
Perceived Self-Efficacy (PSS-PW)	−0.0678 ^t^
Swiss-SEP	−0.0048
PSS-PW × SEP	0.0013 *
Age	−0.0137 ***
Education	0.0085 **
Sex	0.0166
R^2^	0.1244

^t^ < 0.10, * *p* < 0.05, ** *p* < 0.01, *** *p* < 0.001.

## Data Availability

The data that support the findings of this study are available from the corresponding author upon request.
